# On the Blink: The Importance of Target-Distractor Similarity in Eliciting an Attentional Blink with Faces

**DOI:** 10.1371/journal.pone.0041257

**Published:** 2012-07-18

**Authors:** Kathrin Müsch, Andreas K. Engel, Till R. Schneider

**Affiliations:** Department of Neurophysiology and Pathophysiology, University Medical Center Hamburg-Eppendorf, Hamburg, Germany; University of Sydney, Australia

## Abstract

Temporal allocation of attention is often investigated with a paradigm in which two relevant target items are presented in a rapid sequence of irrelevant distractors. The term Attentional Blink (AB) denotes a transient impairment of awareness for the second of these two target items when presented close in time. Experimental studies reported that the AB is reduced when the second target is emotionally significant, suggesting a modulation of attention allocation. The aim of the present study was to systematically investigate the influence of target-distractor similarity on AB magnitude for faces with emotional expressions under conditions of limited attention in a series of six rapid serial visual presentation experiments. The task on the first target was either to discriminate the gender of a neutral face (Experiments 1, 3–6) or an indoor/outdoor visual scene (Experiment 2). The task on the second target required either the detection of emotional expressions (Experiments 1–5) or the detection of a face (Experiment 6). The AB was minimal or absent when targets could be easily discriminated from each other. Three successive experiments revealed that insufficient masking and target-distractor similarity could account for the observed immunity of faces against the AB in the first two experiments. An AB was present but not increased when the facial expression was irrelevant to the task suggesting that target-distractor similarity plays a more important role in eliciting an AB than the attentional set demanded by the specific task. In line with previous work, emotional faces were less affected by the AB.

## Introduction

When we allocate attention to a flux of incoming stimuli, awareness for these stimuli is not constant over time but instead fluctuates from moment to moment. In order to study how visual awareness is changing over time during a stream of quickly succeeding information, rapid serial visual presentation (RSVP) paradigms are widely used. In these paradigms, one or more targets have to be reported in a stream of rapidly succeeding stimuli. If two task-relevant targets appear in close temporal proximity within a stream of irrelevant distractors, a period of limited awareness for the second target, called the AB, is often observed. The AB reflects a deficit in reporting the second target (T2) in case it follows the first task-relevant target (T1) with a temporal delay of 100–400 ms [1], [2], [3]. Single task control conditions in this type of experiments suggest an attentional rather than perceptual cause of the AB [2]. In single task conditions physical stimulation remains the same (presentation of T1 and T2) but attentional demands are decreased, as only the second stimulus is task-relevant. In single task conditions the AB is usually absent [2].

Traditional models have attributed the AB to attentional capacity limitations at a late processing stage [4], [5], [6], [7]. In particular, these models suggested that the perceptual representation for the second target T2, formed during an early processing stage, cannot be transferred into working memory, and thus will not be reported, until the system has successfully transferred the first target T1 into working memory at a late processing stage. However, limited capacity models cannot account for some recent findings of the AB [8]. More recent accounts suggest that the AB results from active control of attentional resources [9], [10], [11]. These models are able to explain why salient stimuli can outlive the AB: the encoding of salient stimuli needs fewer resources due to increased bottom-up strength, and thus less allocation of attentional resources is necessary [9], [11]. Saliency can either be driven by perceptual features, such as discernability of targets from distractors, or by contents (e.g., emotional vs. neutral stimuli).

Several studies employed neutral face stimuli for probing the AB achieving mixed results. Most studies found an AB for faces (Table S1), whereas others did not with famous faces [12], low T1 load [13], upright faces [14], or when T1 and T2 were faces [15], [16]. Landau and Bentin [13] suggested that the saliency of faces among nonface distractors was an important factor in determining the susceptibility of face targets to be blinked. However, they did not specifically investigate this claim. Taken together, these results suggest that face processing requires attentional resources and that the perceptual saliency of faces among distractors is critical for eliciting an AB.

The AB magnitude can be modulated by manipulating the allocation of attention towards T1 or T2 [8]. For example, AB magnitude was reduced by task-irrelevant mental performance in an additional memory task or by focusing less on the AB task [17]. The AB was also extinguished when highly familiar or famous faces were used [12]. In addition, emotional target stimuli seem to modulate blink magnitude as well. Several studies have demonstrated an influence of emotional information on the extent of the blink magnitude by using a variety of emotional stimuli including words [18], [19], [20], photographs of objects or scenes [21], [22], [23], [24] and emotional faces [25], [26], [27], [28], [29]. Interestingly, the AB is differentially modulated depending on whether T1 or T2 is emotionally salient. The AB is increased following an emotional T1, possibly due to a longer attentional dwell time on T1, leaving less capacity for the processing of T2 [29], [30]. In contrast, the AB is attenuated when emotional compared to unemotional stimuli are presented as T2, which suggests stronger attentional capture by emotional stimuli [20], [28]. Importantly, several studies found a robust AB for neutral compared to realistic [26], [29] or schematic emotional faces [31], [32].

In contrast to studies reporting an emotional modulation of the AB in healthy individuals [20], [24], [28], several studies reported an emotional modulation of the AB only in individuals with high anxiety scores [27], [33], with dysphoria [34], or with posttraumatic stress symptoms [35], yet failed to find an effect in healthy participants. Such an absence of the AB is unlikely to be caused by the type of stimulus material because similar stimuli were used as in experiments, which found an AB in healthy individuals (e.g., words in [33], [34], [35] and faces in [27]). Amir and colleagues [35] suggested that this absence might be related to the depth of target processing (e.g., semantic processing or explicit emotion processing). Accordingly, in a series of experiments semantic processing [30] or emotion processing [29] were shown to be a necessary condition for an increased AB following emotional stimuli as T1. For emotional T2 it has not yet been investigated systematically whether explicit emotion processing is required to decrease AB magnitude.

The aim of the present study was to systematically investigate the influence of target-distractor similarity. In total, six experiments were conducted in different groups of participants in order to investigate how emotional valence modulates the temporal allocation of attention. As only a shallow AB was elicited in Experiment 1, we selectively manipulated the T1 and T2 similarity (Experiment 2), similarity of targets and distractors (Experiment 3, 4, and 5), and the task relevance of the emotional expression (Experiment 6). Manipulating experimentally the similarity between targets and distractors revealed a strong effect of the type of distractors and accounted for the shallow and missing AB effect of the previous experiments. The final experiment demonstrated that the type of task (whether the emotional expression was explicitly or implicitly task-relevant) did not have an impact over and above the effect of target-distractor similarity in Experiments 3 and 4.

## Experiment 1

### Materials and Methods

#### Ethics Statement

The participants of this and the subsequent experiments provided written, informed consent. All procedures were approved by the ethics committee of the Hamburg Medical Association.

#### Participants

Fifteen participants (10 female, M ± SD = 24.0±2.3 years) were recruited from the University Medical Center Hamburg-Eppendorf and were paid for participation. All participants had normal or corrected to normal vision and normal color vision [36] and reported no history of psychiatric or neurological illness. One male participant had to be excluded due to performance at chance level.

#### Stimuli

**Figure 1:**
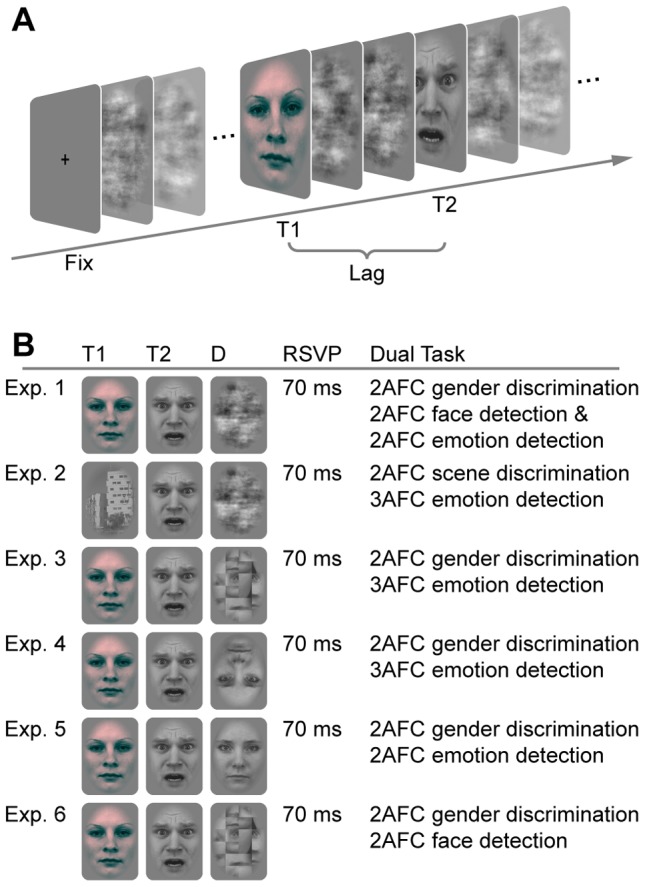
Illustration of a single trial and overview of experiments. (A) After 500 ms fixation period, 25 stimuli including the two targets with a variable lag were rapidly presented (lag 3 in this example). The first *and* the second target were task-relevant. T1 was presented between position 9 and 15 in a stream of distractors followed by T2 at lags 1, 2, 3, 4, 5, 6, 8. (B) The experiments differed with regard to stimuli used as T1, T2, and distractors, and dual task demands. Abbreviations: Fix, fixation; T1, first target; T2, second target; D, distractors; RSVP, rapid serial visual presentation; Exp., experiment; 2AFC, two-alternative forced-choice.

Emotional and neutral faces were embedded among distractors in a RSVP stream (Figure 1). Faces of 12 males and 12 females with neutral, fearful, and happy expressions from the Karolinska Directed Emotional Faces (KDEF; [37]) served as targets. These faces were selected for highest gender discernability as determined in a pilot rating. Distractors were phase-scrambled versions of 54 neutral faces. All stimuli were converted to gray-scale, matched for luminance and masked by an oval shape to remove hair, neck and background information. T1 faces were presented in a red tint (each pixel value of the red color channel multiplied by 2.25) in order to distinguish it from the other stimuli in the stream.

#### Design and Procedure

Each trial consisted of a stream of 25 visual stimuli including scrambled distractors and target faces, starting with a 500 ms fixation period. Each stimulus was displayed for 70 ms at the center of the monitor, resulting in a stimulation frequency of 14.3 Hz (Figure 1). The first face (T1) always had a neutral expression whereas the expression of the second face (T2) was systematically varied (fearful, happy, and neutral expression 31.7% each; remaining 5% scrambled distractor). The temporal interval between T1 and T2 varied between lag 1 (70 ms, no intervening item between T1 and T2), lag 2 (140 ms, one intervening item and so forth), lag 3 (210 ms), lag 4 (280 ms), lag 5 (350 ms), lag 6 (420 ms), and lag 8 (560 ms) in order to cover the whole AB interval. The gender of the two targets was counterbalanced and two targets never had the same identity in a given trial. T1 appeared equally often at positions 9 to 15 of each stream.

After each trial, participants were first requested to report the gender of T1 (“male”, “female”) and then whether they had seen a second face (T2; “face”, “no face”) by button press on the keyboard with the left or right index finger, respectively. In case of a “face”, participants were asked to indicate whether the face was emotional or neutral (“emotional face”, “neutral face”). This two-step procedure allowed discriminating different levels of processing: face detection versus emotion detection of T2. The response button mapping was counterbalanced across participants. Seven blocks with 60 trials each were presented in random order. In total, 19 trials per condition were presented (7 lags ×3 emotions  = 399 trials). In 5% of the trials T2 was not present and replaced by a scrambled distractor. To familiarize participants with the experimental procedure, 10 practice trials were presented before each experiment. No speeded responses were demanded and participants received no feedback during the experiment. Stimuli were presented on a 22" CRT monitor at a refresh rate of 100 Hz and a viewing angle of approximately 5.4° using the Psychophysics Toolbox (3^rd^ version; [38], [39]) and Matlab 7 (The MathWorks Inc, Natick, MA, USA).

#### Data Analysis

Mean accuracy was calculated for T1 and T2, respectively. T2 report was analyzed contingent on correct T1 report. For the T2 task the percentage of correct responses was calculated as the proportion of detected relative to the total number of trials presenting a face as T2, separately for fearful, happy, and neutral faces. The detection of T2 was considered more relevant to the AB than the emotion detection because the amount of misses per lag directly reflects the impairment of visual awareness. In addition, false alarms were defined as the proportion of “face” responses to the number of T2-absent trials contingent on correct T1 report. Low values of false alarms indicate that participants were able to perform the task correctly. The percentage of correct responses on T1 and T2 report were subjected to a repeated measures analysis of variance (ANOVA) with lag (1, 2, 3, 4, 5, 6, 8) and emotion (fearful, happy, neutral) as separate within-subject factors. In addition, T1 error rates were compared for trials in which both targets had the same versus the opposite gender to check for a possible confusion between T1 and T2 in the gender discrimination task at each lag. Estimates were Greenhouse-Geisser-corrected whenever appropriate. Original degrees of freedom are reported. Five planned orthogonal contrasts were conducted as follow-up analysis: (1) the linear effect of lag; (2) neutral vs. emotional faces; (3) fearful vs. happy faces; (4) the interaction between lag and neutral vs. emotional faces (4), and (5) the interaction between lag and fearful vs. happy faces. Effect sizes were reported as eta-squared, representing the proportion of accounted variance (η2<0.1 =  small effect size; 0.1<η2<0.25 =  medium effect size; η2>0.25 =  large effect size).

### Results

**Figure 2:**
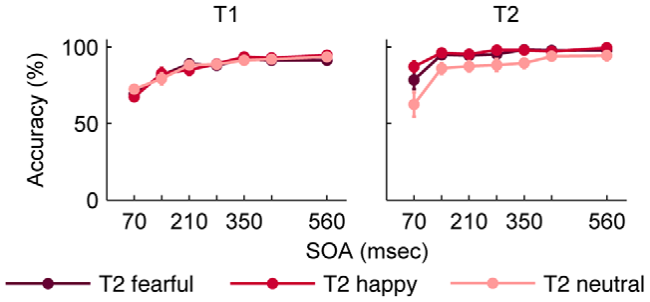
Mean accuracy for T1 and T2 in Experiment 1. Performance is depicted separately for the different facial expressions of T2. T2 detection is conditional on T1 performance. Error bars represent standard errors of the means. Abbreviations: T1, first target; T2, second target; SOA, stimulus onset asynchrony.

In Experiment 1, the comparison of T2 performance in a 7 (lag) x 3 (emotion) repeated measures ANOVA resulted in main effects of lag and emotion and a lag by emotion interaction (Table 1, Figure 2). The contrast analysis on the interaction effect revealed that the effect of lag was more pronounced for neutral faces compared to emotional faces, while the effect of lag was only a trend for the difference of fearful and happy faces (Table 2). The percentage of false alarms was quite low (*M* ± *SD* = 10.5±13.4). These results suggest a temporal impairment of visual awareness modified by emotional expression.

**Table 1:** Results of the repeated measures analysis of variance for each experiment.

	T1				T2			
	*df*	*F*	*p*	*η^2^*	*df*	*F*	*p*	*η^2^*
**Experiment 1 (N = 14)**								
Lag (7)	**6, 78**	**43.21**	**<0.001**	**0.77**	**6, 78**	**11.58**	**0.002**	**0.47**
Emotion (3)	2, 26	0.03	0.975	0.00	**2, 26**	**15.50**	**0.001**	**0.54**
Lag x Emotion	12, 156	0.88	0.565	0.06	**12, 156**	**2.80**	**0.041**	**0.18**
**Experiment 2 (N = 13)**								
Lag (7)	6, 72	0.57	0.756	0.05	6, 72	0.67	0.672	0.05
Emotion (3)	2, 24	0.624	0.454	0.05	2, 24	0.46	0.640	0.04
Lag x Emotion	12, 144	0.98	0.470	0.08	12, 144	0.54	0.884	0.04
**Experiment 3 (N = 21)**								
Lag (7)	**6, 120**	**32.49**	**<0.001**	**0.62**	6, 120	2.65	0.092	0.12
Emotion (3)	2, 40	1.31	0.280	0.06	**2, 40**	**18.50**	**<0.001**	**0.48**
Lag x Emotion	12, 240	1.51	0.175	0.07	12, 240	0.80	0.567	0.04
**Experiment 4 (N = 15)**								
Lag (7)	**6, 84**	**13.28**	**<0.001**	**0.49**	**6, 84**	**6.88**	**0.003**	**0.33**
Emotion (3)	2, 28	0.11	0.893	0.01	**2, 28**	**8.89**	**0.001**	**0.39**
Lag x Emotion	12, 168	1.25	0.292	0.08	12, 168	1.54	0.191	0.10
**Experiment 5 (N = 20)**								
Lag (7)	6, 114	1.77	0.110	0.09	6, 114	0.41	0.738	0.02
Emotion (2)	**1, 19**	**17.03**	**0.001**	**0.47**	**1, 19**	**44.18**	**<0.001**	**0.70**
Lag x Emotion	6, 114	0.94	0.471	0.05	6, 114	0.11	0.995	0.01
**Experiment 6 (N = 16)**								
Lag (7)	**6, 90**	**28.01**	**<0.001**	**0.65**	**6, 90**	**3.73**	**0.038**	**0.20**
Emotion (3)	2, 30	2.26	0.121	0.13	**2, 30**	**13.07**	**0.001**	**0.47**
Lag x Emotion	12, 180	0.62	0.707	0.04	**12, 180**	**2.01**	**0.026**	**0.12**

Abbreviations: T1, first target, T2, second target; *df*, degrees of freedom; *F*, *F*-value; *p*, *p*-value; *η^2^*, effect size; N, sample size.

**Table 2:** Results of the planned contrast analysis for each experiment.

	T1				T2			
	*df*	*F*	*p*	*η^2^*	*df*	*F*	*p*	*η^2^*
**Experiment 1 (N = 14)**								
Contrast 1	**1, 13**	**129.84**	**<0.001**	**0.91**	**1, 13**	**22.18**	**<0.001**	**0.63**
Contrast 2	1, 13	0.05	0.826	0.00	**1, 13**	**16.48**	**0.001**	**0.56**
Contrast 3	1, 13	0.00	0.958	0.00	**1, 13**	**6.03**	**0.029**	**0.32**
Contrast 4	1, 13	1.64	0.223	0.11	**1, 13**	**8.16**	**0.013**	**0.39**
Contrast 5	1, 13	1.16	0.301	0.08	1, 13	3.49	0.084	0.21
**Experiment 2 (N = 13)**								
Contrast 1	1, 12	2.15	0.168	0.15	1, 12	0.96	0.347	0.07
Contrast 2	1, 12	1.93	0.190	0.14	1, 12	0.25	0.625	0.02
Contrast 3	1, 12	0.51	0.489	0.04	1, 12	0.58	0.460	0.05
Contrast 4	1, 12	1.93	0.190	0.14	1, 12	0.04	0.837	0.00
Contrast 5	1, 12	0.38	0.549	0.03	1, 12	2.30	0.155	0.16
**Experiment 3 (N = 21)**								
Contrast 1	**1, 20**	**183,49**	**<0.001**	**0.90**	1, 20	3.26	0.086	0.14
Contrast 2	1, 20	0.29	0.594	0.01	**1, 20**	**23.86**	**<0.001**	**0.54**
Contrast 3	1, 20	2.15	0.159	0.10	**1, 20**	**5.01**	**0.037**	**0.20**
Contrast 4	1, 20	0.07	0.793	0.00	1, 20	1.34	0.260	0.06
Contrast 5	1, 20	3.30	0.084	0.14	1, 20	0.10	0.756	0.01
**Experiment 4 (N = 15)**								
Contrast 1	**1, 14**	**31.24**	**<0.001**	**0.69**	**1, 14**	**9.62**	**0.008**	**0.41**
Contrast 2	1, 14	0.00	0.954	0.00	**1, 14**	**13.30**	**0.003**	**0.49**
Contrast 3	1, 14	0.20	0.661	0.01	1, 14	1.71	0.213	0.11
Contrast 4	1, 14	0.97	0.341	0.07	**1, 14**	**6.97**	**0.019**	**0.33**
Contrast 5	1, 14	0.01	0.935	0.00	1, 14	1.53	0.236	0.10
**Experiment 5 (N = 20)**								
Contrast 1	1, 19	1.63	0.217	0.08	1, 19	0.29	0.598	0.02
Contrast 2	-	-	-	-	-	-	-	-
Contrast 3	**1, 19**	**17.03**	**0.001**	**0.47**	**1, 19**	**44.18**	**< 0.001**	**0.70**
Contrast 4	-	-	-	-	-	-	-	-
Contrast 5	**1, 19**	**4.38**	**0.05**	**0.19**	1, 19	0.16	0.691	0.01
**Experiment 6 (N = 16)**								
Contrast 1	**1, 15**	**82.45**	**<0.001**	**0.85**	**1, 15**	**6.72**	**0.020**	**0.31**
Contrast 2	1, 15	4.19	0.059	0.22	**1, 15**	**22.74**	**<0.001**	**0.60**
Contrast 3	1, 15	0.59	0.453	0.04	1, 15	0.001	0.972	0.00
Contrast 4	1, 15	0.00	0.968	0.00	1, 15	3.65	0.075	0.20
Contrast 5	1, 15	0.02	0.885	0.00	1, 15	0.71	0.413	0.05

Note: Contrast 1 tests for a linear trend on the factor lag. Contrast 2 compares neutral vs. emotional faces and contrast 3 fearful to happy faces. Contrasts 4 and 5 investigate a linear trend on the factor lag for neutral vs. emotional and fearful vs. happy faces, respectively. Abbreviations: T1, first target, T2, second target; *df*, degrees of freedom; *F*, *F*-value; *p*, *p*-value; *η^2^*, effect size; N, sample size.

T1 performance was compared in a 7 (lag) x 3 (emotion) repeated measures ANOVA. The correct report of T1 was dependent on the lag (Table 1, Figure 2), which was reflected by a linear increase across lags (Table 2).

T1 error rates for trials in which T1- and T2-faces had opposite sex were higher compared to trials in which T1- and T2-faces were the same sex only at lag 1 (opposite sex *M* ± *SD* = 14.5±11.0, same sex *M* ± *SD* = 46.7±12.3; *t*_13_ = 6.34, *p*<0.001) but not at any other lag (all *t*s<1.33, all *p*s>0.205; except for lag 3, *t*_13_ = 2.20, *p* = 0.046, not significant following Bonferroni correction).

### Discussion

The decreased performance on T2 could be interpreted as a genuine AB, which additionally was modulated by emotional expression. However, the profile of the AB was very shallow.

Performance on T1 was also reduced in the first two lags. Participants may have confused T1 and T2 at shorter lags, especially when there was no distractor in between. An additional analysis on T1 errors revealed preliminary evidence for this assumption: error rates for opposite-sex compared to same-sex trials were only higher at lag 1 but not at any other lag. Thus, it seems likely that participants confused T2 and T1 in the gender classification task. Earlier studies using letters also found increased order inversion effects for T1 at the first lag [4], [40], [41]. According to the 2-stage competition model [5] there is a trade-off between T1 and T2 performance when the lag between the targets is less than 100 ms. Hence, it seems inherent in the AB that correct report of T1 is compromised by correct report of T2 at the first lag [41]. However, the present results merely reflect a globally diminished performance for T1 and T2 instead of a trade-off between targets.

## Experiment 2

To rule out the possibility that participants confused T2 with T1 stimuli in the gender classification task, neutral T1-faces were replaced by indoor and outdoor scenes in Experiment 2.

### Materials and Methods

#### Participants

Thirteen students (8 female, M ± SD = 24.1±1.5 years), none of whom participated in Experiment 1, were recruited from the same pool and were paid for participation. All participants had normal or corrected to normal vision and reported no history of psychiatric or neurological illness.

#### Stimuli

Stimuli were identical to those of Experiment 1 except that gray-scale indoor and outdoor scenes instead of neutral faces were presented as T1. T1 scenes were not tinted because they could easily be discriminated from T2 faces (compare [26]). Visual scenes (equal in mean luminance) were selected according to highest discrimination performance and matched for visual complexity according to a pilot rating.

#### Design and Procedure

Unlike in the previous experiment, the task on T2 consisted of only one question. An additional response option for “no face” was included, thus resulting in three response possibilities (“emotional face”, “neutral face”, “no face”) for each trial.

#### Data Analysis

Data analysis was identical to Experiment 1 except for the following changes. False alarms in Experiments 2, 3, and 4 were defined as the proportion of “emotional face” or “neutral face” responses to the number of T2-absent trials contingent on correct T1 report.

### Results

**Figure 3:**
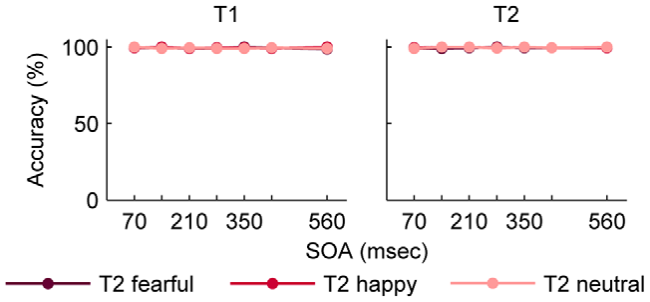
Mean accuracy for T1 and T2 in Experiment 2. Performance is depicted separately for the different facial expressions of T2. T2 detection is conditional on T1 performance. Error bars represent standard errors of the means. Abbreviations: T1, first target; T2, second target; SOA, stimulus onset asynchrony.

T1 performance and T2 performance were separately subjected to a 7 (lag) x 3 (emotion) within-subjects ANOVA. There were no significant effects on T1 performance and on T2 performance (Table 1, Table 2, Figure 3). As in the previous experiments, the percentage of false alarms was low (*M* ± *SD* = 2.2±4.2).

### Discussion

Surprisingly, there were no effects of lag or emotion in Experiment 2 suggesting that the transient performance decrease in Experiment 1 resulted from a confusion of the target faces [4], [5], [11], [40], [41]. The absence of an AB is in direct contrast to the study by De Martino and colleagues [26] who reported an AB also using scenes as T1, faces as T2, and scrambled distractors. In their experiment performance for fearful T2 faces was higher than for neutral T2 faces only at lag 5 (350 ms), which was, however, the only lag tested in this experiment. The distractors in the experiment by De Martino and colleagues [26] differed from the ones in the present Experiments 1 and 2. The role of distractors in eliciting an AB for faces was addressed in the following three experiments.

## Experiment 3, Experiment 4, & Experiment 5

In contrast to previous studies using upright neutral faces [27], 180° rotated neutral faces [25], or randomly rearranged parts of face or scene images [26], [28] as distractors, the distractors in the present Experiments 1 and 2 were phase-scrambled versions of the face stimuli and contained no meaningful high-level information. A previous study using letters reported that the AB could be eliminated when targets were embedded in highly discriminable distractors [4]. To investigate whether the shallow AB profile in Experiment 1 might have resulted from insufficient masking and from dissimilarity between targets and distractors, the similarity of the distractors with the target faces was varied in the following three experiments. They are reported together because every participant took part in two of the experiments.

### Materials and Methods

#### Participants

Twenty-eight participants (15 female, M ± SD = 26.5±4.0 years), none of whom participated in the previous Experiments 1 and 2, were recruited from the same pool and were paid for participation. All participants had normal or corrected to normal vision and reported no history of psychiatric or neurological illness.

#### Stimuli

Target stimuli were identical to those of Experiment 1. Phase-scrambled distractors were replaced by three different types of distractors of the same 54 neutral faces resulting in three experiments. In Experiment 3, faces were divided into 20 randomly rearranged parts of 75×70 pixels and masked by an oval shape to remove hair, neck and background information. These distractors will be referred to as mosaic-scrambled faces. In Experiment 4, distractors consisted of 180° rotated faces with neutral expression. In Experiment 5, distractors were upright faces with neutral expression.

#### Design and Procedure

Design and procedure were identical to that of Experiment 1 except for the following specifications: each participant took part in two experiments. The order of the experiments was counterbalanced across subjects resulting in final samples of 21 participants in Experiment 3 (12 female, M ± SD = 26.6±4.2 years), 15 participants Experiment 4 (8 female, M ± SD = 26.8±2.9 years), and 20 participants Experiment 5 (10 female, M ± SD  = 26.0±4.5 years). In Experiments 3 and 4 the task on T2 was identical to that of Experiment 2 providing three response options in each trial (“emotional face”, “neutral face”, “no face”). In Experiment 5, T2 was always present resulting in a total of 399 trials (7 lags x 3 emotion, 19 trials per condition). The T2 task remained an emotion detection task. However, since distractors were upright neutral faces, the option “no face” was inappropriate for Experiment 5 and only two of the previous response options were provided (“emotional face”, “neutral face”). Hence, participants replied with “neutral face” when they did not see an emotional face in a given trial.

#### Data Analysis

Data analysis for the three experiments was identical to that of Experiment 1 except for Experiment 5 using neutral face distractors, in which the percentage of correct responses for the T2 task was calculated as the proportion of correct emotion detection. Only fearful and happy T2 were analyzed, as neutral T2 could not be differentiated from distractors and faces were always present as distractors. In Experiment 5, false alarms were calculated as the proportion of “emotional face” responses to the number of trials depicting neutral T2 faces contingent on correct T1 report. In addition, these false alarm rates were compared to hit rates for “emotional face” responses in order to clarify whether the absent AB was due to a floor effect.

### Results

**Figure 4:**
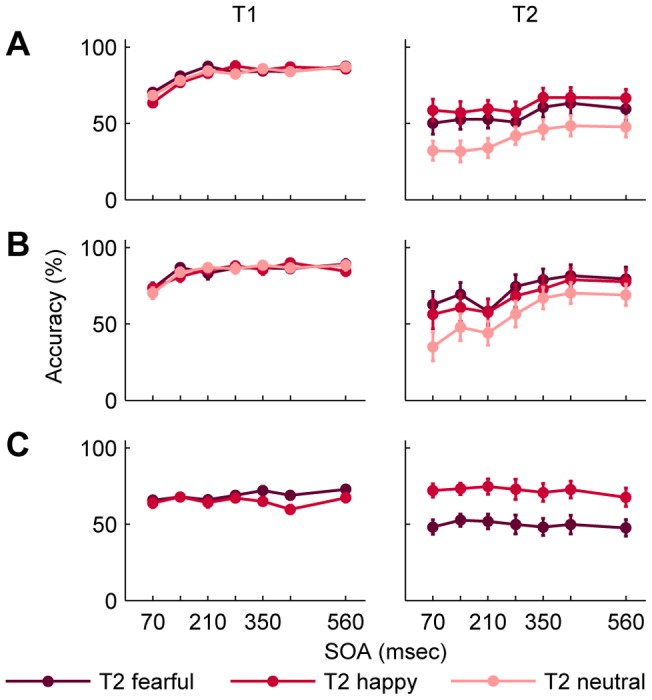
Mean accuracy for T1 and T2 in Experiment 3, 4, and 5. Target stimuli were identical to Experiment 1. Distractors were either (A) mosaic-scrambled faces, (B) inverted faces with neutral expression, or (C) upright faces with neutral expression. Performance is depicted separately for the different facial expressions of T2. T2 performance is conditional on T1 performance. Error bars represent standard errors of the means. Abbreviations: T1, first target; T2, second target; SOA, stimulus onset asynchrony.

For Experiment 3 using mosaic-scrambled face distractors, T1 performance and T2 performance were separately compared in a 7 (lag) x 3 (emotion) within-subjects ANOVA. The ANOVA on T2 performance resulted in main effects of lag and emotion (Table 1, Figure 4A). The contrast analysis showed that the difference between neutral and emotional faces was larger than that between the two emotional faces (Table 2). Although the difference between emotional and neutral faces seemed to be greater at early relative to late lags, the interaction effect did not reach significance. Correct report of T1 depended on lag (Table 1, Figure 4A), which was reflected by a linear increase across lags (Table 2). These results indicate that an AB was found for faces, which was not modulated by emotional expression. However, performance for emotional faces was better than for neutral faces across all lags.

For Experiment 4 presenting inverted face distractors, T1 performance and T2 performance were separately subjected to a 7 (lag) x 3 (emotion) within-subjects ANOVA. Comparison of T2 performance resulted in a main effect of lag and emotion (Table 1, Figure 4B). Follow-up analysis suggested a linear increase across lags and easier detection of emotional compared to neutral faces (Table 2). Notably, although the interaction effect did not reach statistical significance, the planned interaction contrast for the comparison of neutral to emotional faces was significant (Table 2), reflecting that the AB for neutral faces was more pronounced relative to emotional faces. Correct report of T1 depended on lag (Table 1, Figure 4B) reflected by a linear increase across lags (Table 2). These results suggest a transient impairment of visual awareness and an advantage for the detection of emotional faces.

For Experiment 5 using neutral face distractors, T1 performance and T2 performance were separately subjected to a 7 (lag) x 2 (emotion) within-subjects ANOVA. Only effects of emotion were found which were opposite for T1 and T2 performance (Table 1, Table 2, Figure 4C): T1 was reported correctly more often when it was followed by a fearful instead of a happy face, while T2 performance was higher for happy faces compared to fearful faces. These results indicate that performance differed according to the emotional expression, but no AB was found in Experiment 5.

The percentage of false alarms for T2 was *M* ± *SD* = 10.5±13.3 in Experiment 3 and *M* ± *SD*  = 12.2±14.4 in Experiment 4. For Experiment 5, the percentage of false alarms, reflected by the proportion of “emotional face” responses to the number of T2 trials containing neutral faces, was *M* ± *SD* = 18.9±12.3. This rate was significantly lower than the average number of correct responses for emotional T2 (*M* ± *SD* = 60.3±17.0; *t*_19_ = 9.54, *p*<0.001).

### Discussion

As expected, increasing the similarity of distractors and targets in terms of facial features decreased the overall T2 performance. Importantly, the use of more similar distractors resulted in an AB when distractors were mosaic-scrambled and inverted faces, hence containing more feature information than the abstract phase-scrambled distractors used before. Therefore we conclude that dissimilarity between targets and distractors can account for the missing AB in Experiments 1 and 2.

There was no AB when distractors were upright faces. The absence of an AB with upright face distractors is in direct contrast to the experiment by Fox and colleagues using upright neutral faces as distractors [27]. Longer stimulus duration (110 ms) could account for the higher performance in Fox et al. [27]. However, T1 performance in Experiment 5 was lower than that of Experiments 3 and 4 and of [27]. In addition, performance for fearful faces in the T2 task was almost at chance level. In addition, T1 stimuli and T1 task were different (Table S1): flower T1 had to be discriminated from mushroom T1 [27], thus facilitating the T1 differentiation from T2 stimuli as well as from distractors. These results suggest that the task of Experiment 5 was more demanding than that of the previous experiments and that of [27]. However, participants were able to reliably detect emotional faces from the stream of neutral distractors, as reflected by significantly more hits than false alarms for “emotional face” responses. Thus, results of Experiment 5 corroborate the finding that faces with emotional expressions are spared the AB.

In line with results from Experiment 1, T2 performance depended on the emotional content of T2 suggesting a facilitated processing of fearful and happy faces over neutral faces. A superiority effect for happy faces was found except for the inverted face experiment, in which fearful faces tended to be better recognized than neutral faces. These results are in line with the assumption of enhanced bottom-up attention for emotional stimuli [42], [43].

As in Experiment 1, a decreased T1 performance at lag 1 in the mosaic-scrambled and the inverted face experiment reflected the competition for attentional resources of T1 with T2 at lag 1 [4], [40], [41]. T1 performance in the experiment with upright face distractors was greatly reduced across all lags. In this case upright T1 faces differed from the distractors only in color (red tint) and therefore may have been more difficult to extract from the RSVP stream. Thus, it is likely that participants reported the gender of neighboring faces instead that of T1.

## Experiment 6

This final experiment investigated whether the specific attentional set, i.e. the allocation of attentional resources that is adjusted by the observer (top-down control), had an additional impact on the AB over and above the effect of target-distractor similarity. In contrast to all previous experiments, in which emotion recognition was explicitly demanded by the T2 task, in Experiment 6 the emotional expression of faces was irrelevant to the T2 task. For emotionally expressive T2, the influence of the type of task has never directly been investigated so far. Milders and colleagues [28] successfully elicited an AB with a very similar design but an implicit emotion recognition task.

### Materials and Methods

#### Participants

Seventeen participants (10 female, M ± SD = 28.4±4.1 years), none of whom participated in the previous experiments, were recruited from the same pool and were paid for participation. All participants had normal or corrected to normal vision and reported no history of psychiatric or neurological illness. One female subject had to be excluded due to performance at chance level.

#### Stimuli

Stimuli were identical to those in Experiment 3.

#### Design and Procedure

Design and procedure were identical to that of Experiment 1 except for the task on T2. Participants were solely requested to report whether they had seen an upright second face (“yes”, “no”).

#### Data Analysis

Data analysis was identical to that of Experiment 1.

### Results

**Figure 5:**
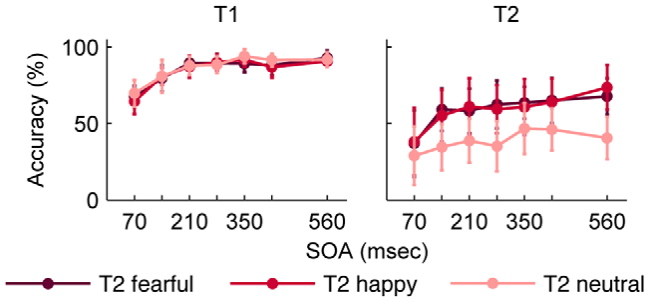
Mean accuracy for T1 and T2 in Experiment 6. Performance is depicted separately for the different facial expressions of T2. T2 detection is conditional on T1 performance. Error bars represent standard errors of the means. Abbreviations: T1, first target; T2, second target; SOA, stimulus onset asynchrony.

The comparison of T2 performance in a 7 (lag) x 3 (emotion) within-subjects ANOVA resulted in main effects of lag, emotion, and an interaction between lag and emotion (Table 1, Figure 5). Follow-up contrast analysis on the interaction revealed a trend in that the linear effect of lag was more pronounced for neutral compared to emotional faces (Table 2). The percentage of false alarms was *M* ± *SD* = 7.6±9.7. These results indicate that an AB was found for faces, which was modulated by emotional expression.

The 7 (lag) x 3 (emotion) within-subjects ANOVA on T1 performance revealed a significant effect of lag (Table 1, Figure 5), which was reflected by a linear increase across lags (Table 2).

### Discussion

Unlike in the previous experiments, the detection of the emotional facial expression of T2 was not relevant to solve the task in Experiment 6. In line with results from Milders and colleagues [28] an AB was observed. The trend of the interaction contrast suggested that the AB was attenuated for happy and fearful faces. Somewhat surprisingly, T2 performance for neutral faces did not recover to baseline level. Reasons for this might be twofold. First, AB patterns in the individual participants were highly heterogeneous (Figure S1). Second, T2 performance for neutral faces seemed to drop particularly from lag 6 to lag 8 (Figure S1; cf. subjects 6, 8, or 11), which most likely reflects an expectation effect: because lag 7 was omitted, participants might not have expected a second face anymore. Emotional but not neutral T2 at lag 8 were detected due to increased saliency. Results of Experiment 6 suggest that the attentional set or the demands of top-down control in the specific task do not have an incremental effect on eliciting the AB beyond the effect of target-distractor similarity of the previous experiments.

## Discussion

The major goal of the present study was to systematically investigate the impact of target-distractor similarity under conditions of high attentional demands in a RSVP stream in which two targets were embedded. Contrary to our expectation, only a shallow AB was found in Experiment 1. However, this effect could not be replicated when we replaced neutral T1 faces by indoor and outdoor scenes in Experiment 2. To investigate whether the absence of an AB resulted from target-distractor dissimilarity and insufficient masking, Experiments 3, 4, and 5 selectively manipulated the distractors' similarity to the target faces. An AB was revealed in Experiment 3 using mosaic-scrambled distractors and Experiment 4 using inverted face distractors. Thus, similarity between targets and distractors seems to account for the strength of the AB in the present experiments. No AB was found, however, in Experiment 5, when targets were emotional faces and distractors were neutral faces. This result supports the notion that faces with emotional expression tend to be less likely to be blinked. Moreover, in Experiments 4 and 6 emotional faces were found to be less susceptible to the AB, further confirming the attentional advantage for emotional faces.

In the first two experiments, the nature of the abstract phase-scrambled distractors and their featural dissimilarity to the targets may have diminished appropriate masking of the target faces. Phase-scrambled distractors may not be sufficiently meaningful or may not contain enough high-level pattern information to function as effective masks. However, previous studies revealed that masks neither have to be meaningful [44] nor have to contain pattern information [45] to be effective. Landau and colleagues [13] suggested that the saliency of faces among nonface distractors was an important factor in determining the susceptibility of face targets to be blinked. Previous studies showing an AB effect on emotional T2 faces used a stream of neutral faces [27], 180° rotated neutral faces [25], or mosaic-scrambled distractors [26], [28] consisting of randomly rearranged parts of faces or scenes. Therefore, the masking effect on T1 by the subsequent distractors may have been stronger in previous studies using faces as targets [25], [26], [27], [28] resulting in larger attentional impairments for processing of T2 compared to our Experiments 1 and 2. This assumption is consistent with a series of AB experiments investigating the role of T1 and its subsequent item in the RSVP stream [46]. The authors reported a correlation between T1 performance and AB magnitude using letters as targets and concluded that masking influenced the AB deficit indirectly by increasing the processing load of T1. Furthermore, Jannati and colleagues (Experiment 2 in [47]) successfully elicited an AB for letters by increasing target-distractor similarity relative to a report using the same experimental design [44], when pseudoletters instead of digits were used as distractors. Our Experiments 3 and 4 also provide support for the role of target-distractor dissimilarity as causes for the missing AB in our first two experiments. The experiments using mosaic-scrambled and inverted face distractors successfully elicited an AB. Using upright neutral faces as distractors resulted in a drop in T1 and T2 performance except for happy faces. However, we did not observe an AB under conditions of minimal target saliency with upright neutral face distractors that were maximally similar to emotionally target faces, supporting the finding that emotional faces tend to outlive the AB. A similar finding of reduced performance without significant AB has also been reported by Awh and colleagues (Experiment 5 in [16]) when faces were masked by other faces. Taken together, the results from Experiments 3 and 4, specifically, corroborate the role of insufficient masking as a cause for the missing and shallow AB in our first two experiments.

Furthermore, results from Experiment 6 suggest that the nature of the (emotion recognition) task does not play a crucial role in shaping the AB over and above the role of target-distractor similarity. Similar to the results of Experiment 3 using an explicit emotion detection task and mosaic-scrambled distractors, an AB was also found in Experiment 6 when participants had to engage in a face detection task on T2, in which the emotional expression of T2 was task-irrelevant. Our result is in line with several other studies reporting an AB with an implicit face detection task [12], [13], [28]. Previous work demonstrated that increasing the task load and changing the instruction had an impact on AB magnitude [17], [48], [49], [50], suggesting that attentional set or top-down control of the specific task plays a role in the elicitation of the AB. However, it did not seem to make a difference for the present experiments, whether the emotional expression was relevant to the task or not.

Face stimuli in the RSVP may be more salient than letters or words and therefore require adequate masks to transiently impair awareness. Faces convey relevant information for social interactions. Several lines of research suggest that face processing differs from processing of other stimuli. Already newborns show increased attention to face compared to nonface stimuli (e.g. [51]). Furthermore, face recognition in contrast to word or object recognition seems to be holistic and configural [52]. Therefore it was hypothesized that faces are processed automatically by a pre-attentive mechanism as they pop out of visual search arrays with different distractors [53]. In addition, faces may be processed with little attentional resources, which is supported by studies showing that faces can be processed in the near-absence of attention [54] or outside of awareness [55], [56]. A recent study found that faces receive mandatory processing during a change detection task [57]. This attentional advantage for faces was still present when additional semantic information was given where to expect the change. These results suggest that even neutral T2 faces receive enhanced attention due to their saliency when presented during the AB interval. Support for this notion comes from several AB studies, which failed to find an AB for neutral faces masked either with nonface stimuli [13], [14], [15], [16] or with other neutral faces [16]. The amygdala has been suggested to be a neuroanatomical key region for the processing of emotionally and socially relevant stimuli [58] and is assumed to contribute to the modulating effect of emotional words on the AB [20]. However, even neutral faces are highly salient and result in increased amygdala activity, and therefore attentional resources may be sufficient to process both target face stimuli irrespective of the emotional expression of T2 in Experiments 1 and 2. Although the majority of studies employing faces as T2 actually found an AB for faces, it is evident that the experimental paradigms reported in the literature are very heterogeneous. Currently it does not seem possible to isolate a single factor or a combination of factors that is able to predict the occurrence or absence of an AB in experiments using face stimuli as targets (Table S1).

In conclusion, our experiments demonstrate that the AB for faces is minimal or absent when targets can be easily discriminated from distractors. When distractors are more similar to target faces, an AB for faces can be reliably obtained. In addition, our results support the notion that the AB is modulated by emotional expression in that neutral faces tend to be blinked more likely than emotional faces.

## Supporting Information

Figure S1 **Mean accuracy for T2 of each participant in Experiment 6.** Performance is depicted separately for the different facial expressions of T2. T2 detection is conditional on T1 performance. Error bars represent standard errors of the means. Abbreviations: T1, first target; T2, second target; SOA, stimulus onset asynchrony.
(TIF)

Table S1 **Studies investigating the AB for T2 face stimuli.** This table summarizes the experimental designs and results of all studies performing an RSVP and presenting faces as T2. Literature search was based on PubMed search terms “attentional blink” and one of the following: “face”, “fear”, “emotion”, or “anxiety”. Abbreviations: T1, first target; T2, second target; SOA, stimulus onset asynchrony; ISI, interstimulus interval; RSVP, rapid serial visual presentation; AB, Attentional Blink; FE, fearful; HA, happy; NE, neutral; 2AFC, 2 alternatives forced choice; SA, sad.
(PDF)
