# Supplementary figures and images for: On the Blink: The Importance of Target-Distractor Similarity in Eliciting an Attentional Blink with Faces
Source: PLoS One. 2012 Jul 18;7(7):e41257. doi: 10.1371/journal.pone.0041257 (PMC3399797; doi:10.1371/journal.pone.0041257)

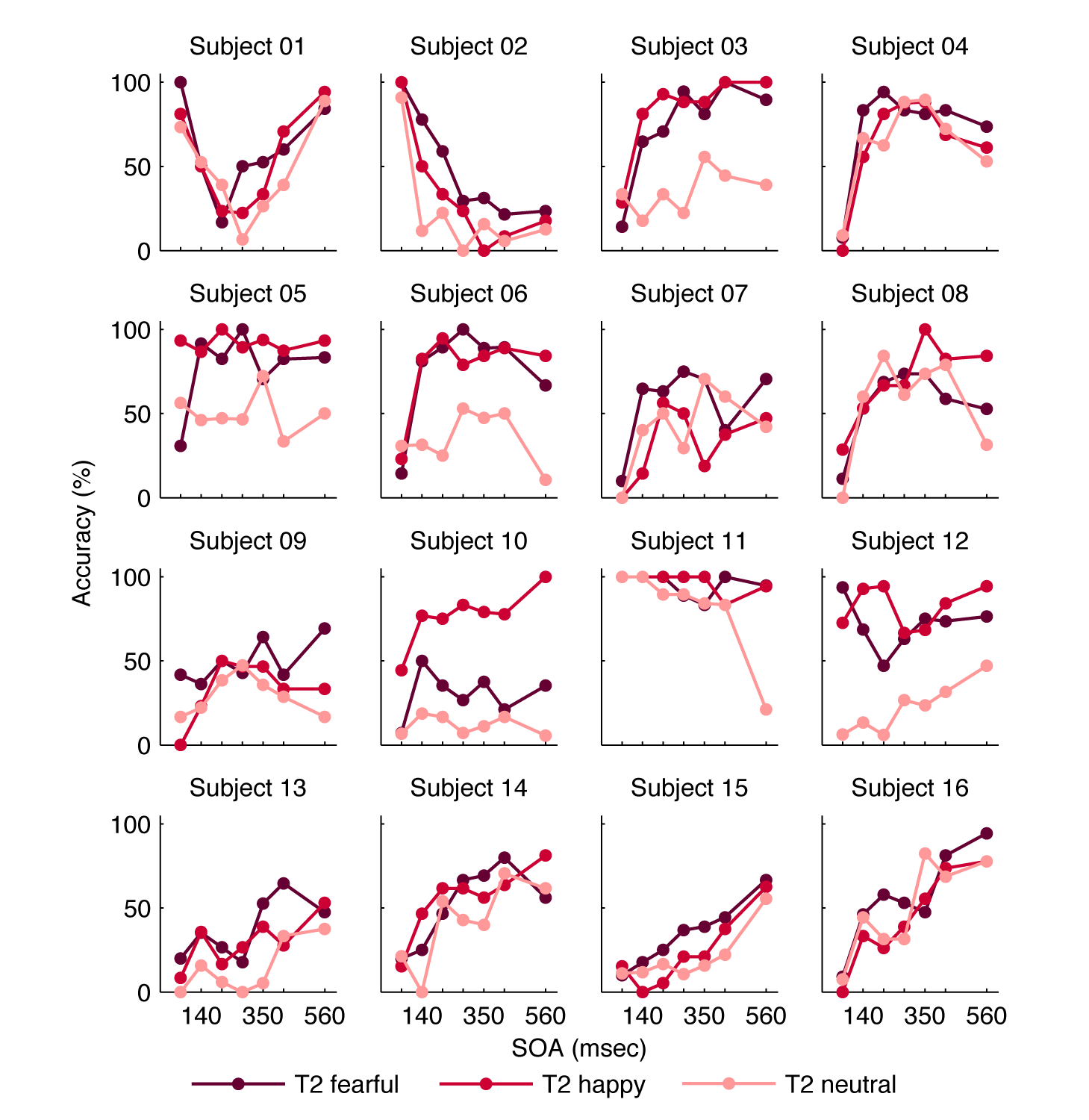

Supplement: Figure S1 — Mean accuracy for T2 of each participant in Experiment 6. Performance is depicted separately for the different facial expressions of T2. T2 detection is conditional on T1 performance. Error bars represent standard errors of the means. Abbreviations: T1, first target; T2, second target; SOA, stimulus onset asynchrony. (TIF) [file pone.0041257.s001.tif]
