# Supplementary material for: On the Blink: The Importance of Target-Distractor Similarity in Eliciting an Attentional Blink with Faces
Source: PLoS One. 2012 Jul 18;7(7):e41257. doi: 10.1371/journal.pone.0041257 (PMC3399797; doi:10.1371/journal.pone.0041257)
Supplement: Table S1 — Studies investigating the AB for T2 face stimuli. This table summarizes the experimental designs and results of all studies performing an RSVP and presenting faces as T2. Literature search was based on PubMed search terms “attentional blink” and one of the following: “face”, “fear”, “emotion”, or “anxiety”. Abbreviations: T1, first target; T2, second target; SOA, stimulus onset asynchrony; ISI, interstimulus interval; RSVP, rapid serial visual presentation; AB, Attentional Blink; FE, fearful; HA, happy; NE, neutral; 2AFC, 2 alternatives forced choice; SA, sad. (PDF) [file pone.0041257.s002.pdf]

Table S1. Studies investigating the AB for T2 face stimuli

	Stimuli			Task								
Name	T1	T2	Distractors/Masks	T1	T2	SOA (ms)	T2/trials (%)	Duration (ms)	ISI (ms)	RSVP length	Size (°)	Main findings
Awh et al., 2004 [1]	3 numbers (experiment 2); 3 female faces (experiment 6)	3 male faces	patterns (for numbers), mosaic-scrambled faces (for faces) presented for 59 ms	3AFC number identification	3AFC face identification	0, 59, 118, 176, 236, 294, 353, 412, 529, 706	100	71	-	4[1]	5.5x4	numbers as T1: no AB; faces as T1: AB
Fox et al., 2005 [2]	flowers, mushrooms	FE, HA faces	NE faces	2AFC flower discrimination	2AFC emotion detection	220, 330, 440, 550, 660, 770	63	110	-	15	n/a	AB for low anxious participants, attenuated for fearful faces in high anxious participants
Jackson & Raymond, 2006 [3]	10 square, 10 circle patterns	1 unfamiliar face	unfamiliar faces	2AFC pattern discrimination	2AFC face detection	85, 170, 255, 340, 425, 510, 595, 680	50	85	-	15	2.9x3.4	AB for unfamiliar but not familiar faces irrespective of familiarity of distractors
Milders et al., 2006 [4]	12 green-tinted FE, 12 green-tinted NE faces	12 FE, 12 NE faces	24 mosaic-scrambled faces	2AFC gender discrimination	2AFC face detection	160, 240, 400, 560	50	80	-	22	15.6x19.7	AB for neutral faces, attenuated for fearful faces
Einhäuser et al., 2007 [5]	faces[2], watches	faces, watches	face	2AFC face and/or watch identification	2AFC face and/or watch identification	depending on RSVP frequency[3]	100	25, 33, 50, 67, 83, 67	-	5 sec	6x6	AB
de Jong & Martens, 2007 [6]	48 AG, 48 HA faces	48 AG, 48 HA faces	299 180° rotated NE faces	3AFC number of faces	2AFC emotion discrimination	240, 360, 960	100	120	-	16	531x720 px	AB, modulated by facial expression
Ryu & Chaudhuri, 2007 [7]	40 houses	6 highly distinct, 6 typical faces	mosaic-scrambled houses and faces	3AFC house identification	3AFC face identification	100, 200, 300, 400, 500, 600, 700, 800	100	100	-	10	7x8.5	AB for distinct and typical faces
Landau & Bentin, 2008 [8]	60 tulips, 60 sunflowers	115 faces, 115 watches	240 furniture objects	2AFC flower discrimination	2AFC T2 detection	79, 237, 553	50	79	-	20	3x3.2	AB for watches but not faces (experiment 1); small AB for faces when T1 also faces (experiment 2); AB for faces when T1 load increased (experiment 4)
Maratos et al., 2008 [9]	1 AG, 1 HA, 2 NE schematic faces	1 AG, 1 HA, 2 NE schematic faces	feature-scrambled schematic faces	2AFC number of faces	3AFC emotion discrimination of last face viewed	257, 386, 514, 643, 771, 1028, 1157	75	129	-	20	5.7x7.5	AB for neutral faces, attenuated for fearful and happy faces between 257 and 386 ms
Sy & Giesbrecht, 2008 [10]	NE, HA, FE faces, pink frame	NE, HA, FE faces, green frame	NE, HA, FE faces, grey frame	2AFC gender discrimination	2AFC gender discrimination	480, 1120	100	80	80	15	4.2x2.9	AB, modulated by target-target similarity
De Martino et al., 2009 [11]	indoor, outdoor scenes	FE, NE faces	mosaic-scrambled scenes and faces	2AFC scene discrimination	3AFC face identification	350	100	70	-	15	8.5x8.5	AB for neutral faces, attenuated for fearful faces
Ganis & Patnaik, 2009 [12]	1 male familiar face (Tom Cruise)	1 male unfamiliar face	15 male unfamiliar faces	none	2AFC face detection	200, 600, -200	50	100	-	16	10x14	AB
Raymond & O'Brian, 2009 [13]	10 square, 10 circle patterns	12 value-laden male faces, 24 novel male faces	20 mosaic-scrambled faces as masks	2AFC pattern discrimination	old/new decision	200, 800	100	85	-	4[4]	2.9x3.6	AB for loss-associated but not win-associated faces of prior value learning task
Serences et al., 2009 [14]	3 numbers (experiment 2); 3 faces of opposite gender than T2 (experiment 3)	3 faces	mosaic-scrambled faces presented for 59 ms	3AFC number or face identification[5]	3AFC face identification	0, 59, 118, 176, 236, 294, 354, 412, 472, 529	100	47 (T1), variable (T2)	-	3[1]	5.5x4	numbers as T1: no AB; faces as T1: AB
Miyazawa & Iwasaki, 2010 [15]	5 flower symbols	3 face icons	symbols	5AFC symbol identification	4AFC face discrimination	79, 140, 210, 350, 560	75	70	-	16	2x2	AB for neutral and fearful faces, attenuated for happy faces
Srivastava & Srinivasan, 2010 [16]	2 letters	4 HA, 4 SA faces	random lines presented for 200 ms	2AFC letter identification	2AFC emotion discrimination	0, 100, 200, 400, 600, 900	100	~64 ms (variable)	-	4[1]	4.5x5.9	AB for sad faces, attenuated for happy faces
Stein et al., 2010 [17]	12 green-tinted NE faces flanked by 2 NE faces	12 FE, 12 HA faces	72 mosaic-scrambled NE faces	2AFC gender discrimination of central face	2AFC face detection	166, 581	50	83	-	22	3.9x5.5	AB for happy faces in both load conditions, attenuated for fearful faces in low-load condition
Darque et al., 2011 [18]	10 square, 10 circle patterns	1 upright NE, 1 inverted NE face	lines (for patterns), patches (for faces) as masks	2AFC pattern discrimination	2AFC eye position discrimination	85, 510	100	85	-	4[4]	2.5x3	AB for inverted but not upright faces
Milders et al., 2011 [19]	8 green-tinted NE faces	8 FE, 8 HA, 8 AG faces with directed or averted eye gaze	24 mosaic-scrambled faces	2AFC gender discrimination	2AFC face detection	160, 560	86	80	-	22	11.6x15.7	AB, no modulation by gaze or emotion
Van Dam et al., 2011 [20]	10 NE, 10 HA, 10 FE male faces	10 NE, 10 HA, 10 FE male faces	54 180° rotated NE faces	3AFC emotion discrimination	3AFC emotion discrimination	321, 642	80	107	-	16	8x11.4	AB for neutral faces, attenuated for fearful and happy faces

Abbreviations: T1, first target; T2, second target; SOA, stimulus onset asynchrony; ISI, interstimulus interval; RSVP, rapid serial visual presentation; AB, Attentional Blink; FE, fearful; HA, happy; NE, neutral; 2AFC, 2 alternatives forced choice; SA, sad; Notes: [1] dwell time paradigm: targets presented horizontally and vertically; [2] faces wearing scarves and sunglasses; [3] different RSVP procedure; [4] only four stimuli used: 2 targets, 2 masks, no distractors; [5] in some blocks speeded responses needed

References

1. Awh E, Serences J, Laurey P, Dhaliwal H, van der Jagt T, et al. (2004) Evidence against a central bottleneck during the attentional blink: multiple channels for configural and featural processing. Cognitive Psychology 48: 95-126.
2. Fox E, Russo R, Georgiou GA (2005) Anxiety modulates the degree of attentive resources required to process emotional faces. Cognitive Affective & Behavioral Neuroscience 5: 396-404.
3. Jackson MC, Raymond JE (2006) The role of attention and familiarity in face identification. Perception & Psychophysics 68: 543-557.
4. Milders M, Sahraie A, Logan S, Donnellon N (2006) Awareness of faces is modulated by their emotional meaning. Emotion 6: 10-17.
5. Einhäuser W, Koch C, Makeig S (2007) The duration of the attentional blink in natural scenes depends on stimulus category. Vision Research 47: 597-607.
6. de Jong PJ, Martens S (2007) Detection of emotional expressions in rapidly changing facial displays in high- and low-socially anxious women. Behavior Research and Therapy 45: 1285-1294.
7. Ryu JJ, Chaudhuri A (2007) Differences in attentional involvement underlying the perception of distinctive and typical faces. Perception 36: 1057-1065.
8. Landau AN, Bentin S (2008) Attentional and perceptual factors affecting the attentional blink for faces and objects. Journal of Experimental Psychology: Human Perception and Performance 34: 818-830.
9. Maratos FA, Mogg K, Bradley BP (2008) Identification of angry faces in the attentional blink. Cognition & Emotion 22: 1340-1352.
10. Sy JL, Giesbrecht B (2009) Target-target similarity and the attentional blink: Task-relevance matters! Visual Cognition 17: 307-317.
11. De Martino B, Kalisch R, Rees G, Dolan RJ (2009) Enhanced processing of threat stimuli under limited attentional resources. Cerebral Cortex 19: 127-133.
12. Ganis G, Patnaik P (2009) Detecting concealed knowledge using a novel attentional blink paradigm. Appl Psychophysiol Biofeedback 34: 189-196.
13. Raymond JE, O'Brien JL (2009) Selective visual attention and motivation: the consequences of value learning in an attentional blink task. Psychological Science 20: 981-988.
14. Serences J, Scolari M, Awh E (2009) Online response-selection and the attentional blink: Multiple-processing channels. Visual Cognition 17: 531-554.
15. Miyazawa S, Iwasaki S (2010) Do happy faces capture attention? The happiness superiority effect in attentional blink. Emotion 10: 712-716.
16. Srivastava P, Srinivasan N (2010) Time course of visual attention with emotional faces. Attenion, Perception, & Psychophysics 72: 369-377.
17. Stein T, Peelen MV, Funk J, Seidl KN (2010) The fearful-face advantage is modulated by task demands: evidence from the attentional blink. Emotion 10: 136-140.
18. Darque A, Del Zotto M, Khateb A, Pegna AJ (2011) Attentional Modulation of Early ERP Components in Response to Faces: Evidence From the Attentional Blink Paradigm. Brain Topography.
19. Milders M, Hietanen JK, Leppanen JM, Braun M (2011) Detection of emotional faces is modulated by the direction of eye gaze. Emotion 11: 1456-1461.
20. Van Dam NT, Earleywine M, Altarriba J (in press) Anxiety attenuates awareness of emotional faces during rapid serial visual presentation. Emotion.
